# Theoretical Analysis of Wave-Front Aberrations Induced from Conventional Laser Refractive Surgery in a Biomechanical Finite Element Model

**DOI:** 10.1167/iovs.61.5.34

**Published:** 2020-05-20

**Authors:** Lihua Fang, Weiwei Ma, Yan Wang, Yu Dai, Zhaohui Fang

**Affiliations:** ^1^ National Engineering Laboratory for Nondestructive Testing and Optoelectric Sensing Technology and Application, Nanchang Hangkong University, Nanchang, China; ^2^ Tianjin Eye Hospital and Eye Institute, Ophthalmology and Visual Development Key Laboratory, Tianjin Medical University, Tianjin, China

**Keywords:** wave-front aberrations, biomechanical effects, finite element model, refractive surgery, displacement

## Abstract

**Purpose:**

To examine the biomechanical effects-induced wave-front aberrations after conventional laser refractive surgery.

**Methods:**

A finite element model of the human eye was established to simulate conventional laser refractive surgery with corrected refraction from –1 to –15 diopters (D). The deformation of the anterior and posterior corneal surfaces was obtained under the intraocular pressure (IOP). Then, the surface displacement was converted to wave-front aberrations.

**Results:**

Following conventional refractive surgery, significant deformation of the anterior and posterior corneal surfaces occurred because of the corneal biomechanical effects, resulting in increased residual wave-front aberrations. Deformation of the anterior surface resulted in a hyperopic shift, which was significantly increased with the increasing refractive correction. The residual high-order aberrations consisted of spherical aberration, vertical coma, and y-trefoil. Spherical aberration was significantly positively correlated to enhanced refraction correction. The effect of posterior corneal surface on induced wave-front aberration was less than the anterior corneal surface. The IOP slightly affects the postoperative defocus, coma, and spherical aberration. When treatment decentration occurred during the procedure, the hyperopic shift decreased as the eccentricity increased. Treatment decentration had a significant impact on the spherical aberration and the coma. In addition, the ocular tissue elasticity played a key role in hyperopic shift, whereas it had little effect on the other aberrations.

**Conclusions:**

Among the many factors that affect high-order aberrations after conventional laser refractive surgery, the alterations in corneal morphology caused by biomechanical effects must be considered, as they can lead to an increase in postoperative residual wave-front aberrations.

Refractive surgery is a safe and effective surgical method for correcting refractive errors, such as myopia, hyperopia, and astigmatism.^1^ Vision correction is achieved by changing the curvature of the anterior surface of the cornea. LASIK involves the creation of a lamellar corneal flap, lifting of the flap, and ablation of the underlying stromal bed.^2^ However, some clinical data have shown that LASIK could significantly increase the incidence of higher-order aberrations.^3^ In fact, not only laser ablation profiles, but also the cosine effect of laser energy loss can result in an increase of higher-order aberrations. Even wave-front-guided refractive surgery cannot completely prevent the expansion nor the long-term, continuous increase of aberrations. Many studies have demonstrated that the increase in postoperative aberrations may be a result of biomechanical effects.^4^ The tissue ablation of the stromal layer leads to biomechanical changes of the cornea, which in turn affects its shape,^5^ resulting in increased incidence of residual wave-front aberrations. Therefore it is of great clinical significance to explore the biomechanical effects on the residual wave-front aberrations.

The finite element method is a computational tool that can be used to represent the geometric, biomechanical, and biological characteristics of a structure.^6^ Although many studies have focused on the finite element model (FEM) of the cornea, little is known about the whole-eye three-dimensional (3D) model of LASIK, which accounts for the entire surface of the eye. Deenadayalu et al.^7^ studied the effects of corneal elasticity, flap diameter and thickness, and intraocular pressure (IOP) on the refractive changes caused by LASIK corneal flap. Although the corneal topographic data of patients were used for curved surface simulation, the extrapolation of geometric points of the sclera caused obvious deviations.^7^ Another study led by Roy and Dupps^6^ focused on the effects of corneal elasticity on the deformation of the cornea before and after LASIK surgery and established an axisymmetric two-dimensional model of the whole eye. The same group also developed a 3D patient-specific FEM to theoretically compare the corneal stress distribution of LASIK with that of small-incision lenticule extraction (SMILE).^8^ Bao et al.^9^ developed and validated a numerical model of LASIK surgery by integrating the effects of corneal biomechanical behavior. Therefore finite element-based biomechanical models of the eye have become important in predicting the effects of LASIK.

Corneal biomechanical properties are related to structure stability, as well as material properties,^10^^,^^11^ and this relationship has been established by several studies. Woo et al.^12^ obtained the nonlinear material properties of the complete cornea and sclera through experimental measurement, finite element analysis, and axisymmetric mathematical modeling. Bryant and McDonnell^13^ demonstrated that the corneal biomechanical response was nonlinear. Furthermore, Anderson et al.^14^ studied the nonlinear response of the cornea through testing and mathematical analysis and applied the Ogden hyperelastic model to the cornea for simulation analysis. However, none of the earlier mentioned studies considered the material properties of the sclera based on the whole-eye 3D model. It is therefore necessary to consider the influence of the material properties of the cornea and sclera on the biomechanical properties after a patient undergoes LASIK.

Clinical data have shown that LASIK can cause high-order aberrations. Maeda et al.^15^ demonstrated that the corneal ectasia after LASIK showed high-order aberrations dominated by coma on the anterior and posterior surfaces of the cornea. Agarwal et al.^16^ found that, in patients with low myopia astigmatism, spherical aberration and total high-order aberrations increased by 0.085 µm and 0.13 µm, respectively, after wave-front-optimized LASIK. Hu et al.^17^ discovered that the factor of IOP contributed to LASIK acting as a trigger for high-order aberrations, especially spherical aberration. The researchers also proposed that IOP should be integrated as a variable for laser surgery in the new algorithm to control high-order aberrations after LASIK.^17^ In summary, researchers must consider the potentially significant influence of biomechanical factors in their study of corneal refractive surgery, which can be further accurately simulated by finite element analysis.

This study aims to evaluate the biomechanical effects-induced wave-front aberrations after conventional laser refractive surgery. Three-dimensional FEM of human eyes can be used for quantitative analysis of the wave-front aberrations introduced by the biomechanical effects quantification of the postoperative outcomes, and comparison of clinical findings to elucidate the biomechanical effects on higher-order aberrations after refractive surgery. It is not only important for the optimization of preoperative screening and postoperative visual quality, but also provides preventive measures to reduce the risk of iatrogenic corneal ectasia.

## Methods

### FEM of Human Eye

Gullstrand proposed the eye model that established the research basis for future improvement, and it has been recognized as the most widely used optical eye model in the field of optics. The 3D human eye model is based on data obtained using the classic Gullstrand precision model,^18^ which was constructed in the software SIEMENS NX (Siemens PLM Software, Plano, TX, USA).

**Figure 1.**
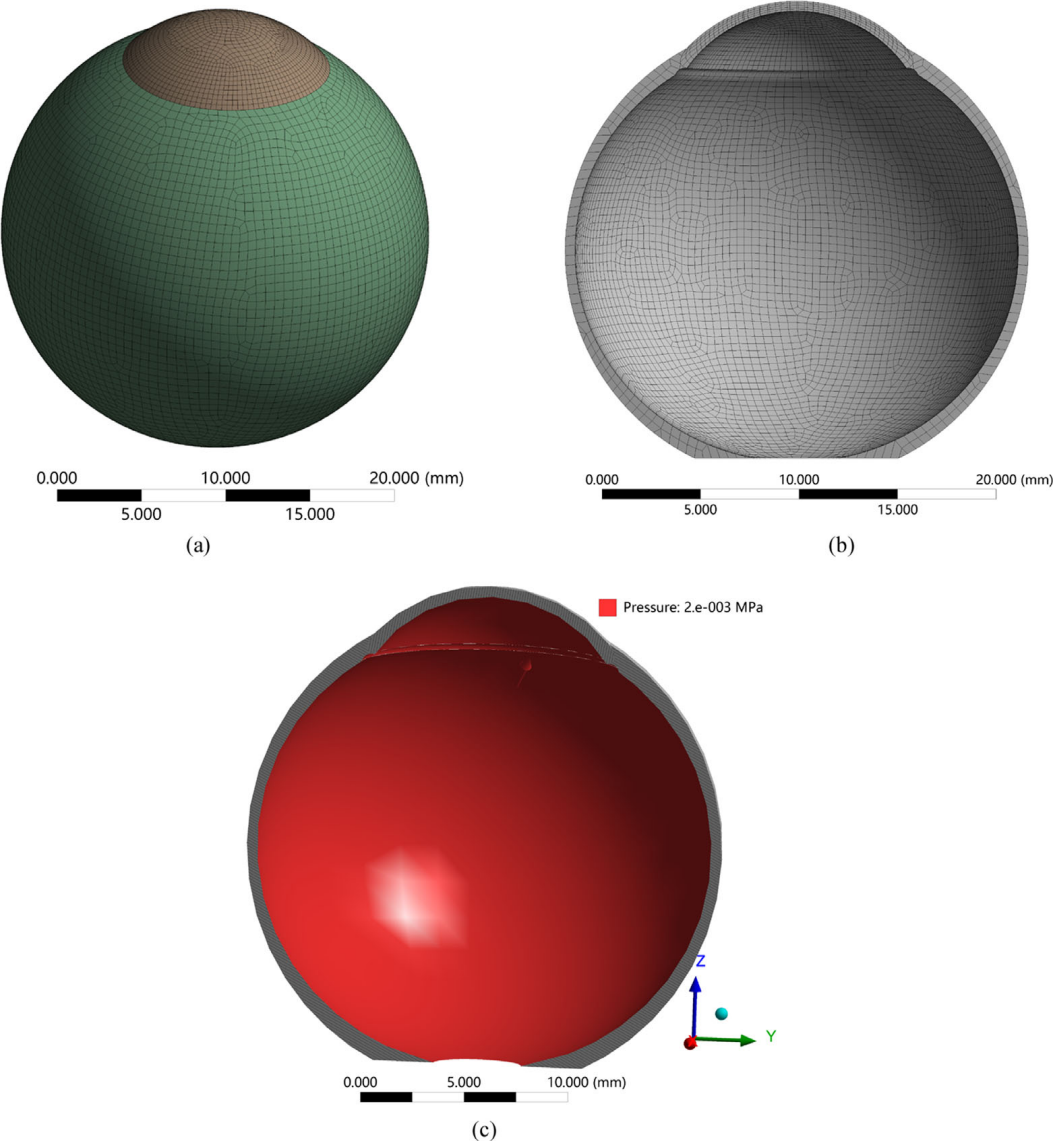
(**a**) The meshed whole-eye model. (**b**) Cross-section view of the 3D eye model meshes. (**c**) The inner surface of the eyeball was subjected to the IOP.

According to the shape and characteristics of the cornea and sclera, we divided them into different-sized hexahedral meshes in the finite element software ANSYS (ANSYS, Canonsburg, PA, USA). We focused primarily on the deformation of the postoperative cornea, whose meshes were relatively thin. To ensure both computational efficiency and simulation accuracy, when mesh density failed to achieve a significant change in nodal displacement, we considered the model sufficiently resolved. Therefore in this model, the numbers of corneal nodes, corneal mesh cells, scleral nodes, and scleral mesh cells were 33654, 9359, 67857, and 18394, respectively. For diverse myopic diopters, the numbers of units and nodes included in the corneal model varied slightly. The meshed human eye model and cross-section view are shown in Figures 1a and 1b.

To prevent rigid-body motion, the models were restrained in posterior scleral nerves, which were surrounded by the optic nerve bundle and other biological tissues. The force of fixation was sufficient for use as the boundary constraint of the research model. Therefore a constrained fixed support had been applied on the bottom of the sclera (the cut-section diameter of the hole was approximately 4 mm). In addition, to avoid the effect of nonphysiological boundary attributed to the cornea alone, the cornea was bonded contact with the sclera to better describe the physiological situation. Meanwhile, a fluid cavity enclosed by the internal surface of the eye globe was modeled and used to simulate the effect of IOP. As shown in Figure 1c, the IOP was applied normal to all inner surfaces of the model.

### Material Properties

A previous study by Woo et al.^12^ demonstrated that the cornea and sclera show nonlinear material properties. The properties of this nonlinear material can be summarized in a hyperelastic material model based on the Ogden strain energy function, which represents the hyperelastic, isotropic, and incompressible features of the cornea and sclera. The strain energy potential can be expressed as follows^19^:

(1) $$W=\sum_{i=1}^{N}\frac{\mu_{i}}{\alpha_{i}}\left({{\bar{\lambda }}_{1}}^{\alpha_{i}}+{{\bar{\lambda }}_{2}}^{\alpha_{i}}+{{\bar{\lambda }}_{3}}^{\alpha_{i}}-3\right)+\sum_{k=1}^{N}\frac{1}{d_{k}}{\left(J-1\right)}^{2k}$$

where *W* is the strain energy potential, ${\bar{\lambda }}_{p}=J^{-\frac{1}{3}}\lambda_{P}$ is the deviatoric principal stretch, *J* is the determinant of the elastic deformation gradient, *λ_p_ *is the principal stretch of the left Cauchy–Green tensor, and *N*, *µ_i_*,* α_i_*, and *d_k_ *are material constants representing the tissue's hyperelasticity and compressibility.

The initial shear modulus µ is defined by:

(2) $$\mu =\frac{1}{2}\sum_{i=1}^{N}\alpha_{i}\mu_{i}$$

The initial bulk modulus *k* is defined by:

(3) $$k=\frac{2}{d_{1}}$$

A higher value of *N* can provide a better fit to the exact solution. It may, however, cause numerical difficulties in fitting the material constants. For this reason, we choose *N* = 2 and *N* = 1 as corneal and scleral fitted parameters. The corneal fitted parameters are as follows: *µ_1_* = 0.003535 Mpa, *α_1_* = 103.51, *µ_2_* = 0.003535 Mpa, and *α_2_* = 103.61. The scleral fitted parameters are as follows: *µ_1_* = 0.030224 Mpa, and *α_1_* = 182.73. In addition, *d_1_* is set to 0 to account for the near incompressibility of the cornea and sclera.

### Simulation of Conventional Refractive Surgery

Because the cornea is set as a geometric sphere, only pure myopia or hyperopia components are included in the refractive errors of the whole eye. Based on the Munnerlyn equation, the ablation profile was proposed for calculating the ablation depth of the cornea.^20^ The ablation depth of the cornea for myopic correction is given by:

(4) $$l\left(d\right)=\sqrt{R_{1}^{2}\;-\;d^{2}}\;-\;\sqrt{R_{f}^{2}\;-\;d^{2}}+\sqrt{R_{f}^{2}\;-\;{\left(O/2\right)}^{2}}\;-\;\sqrt{R_{1}^{2}\;-\;{\left(O/2\right)}^{2}}$$

Here, *d* is the distance of any arbitrary point in the pupil plane to the center of the pupil, and *R_1_* represents the radius of curvature of the anterior corneal surface before refractive surgery. *R_f_* conveys the radius of curvature after refractive surgery. *O* is the diameter of the optical zone. *R_f_* can be obtained as follows:

(5) $$R_{f}=\frac{1000\left(n-1\right)R_{1}}{\left(n-1\right)+D_{s}R_{1}}$$

Here, *D_s_* depicts the myopic (hence negative) refraction in diopters; *n* represents the refractive index of the cornea.

**Figure 2.**
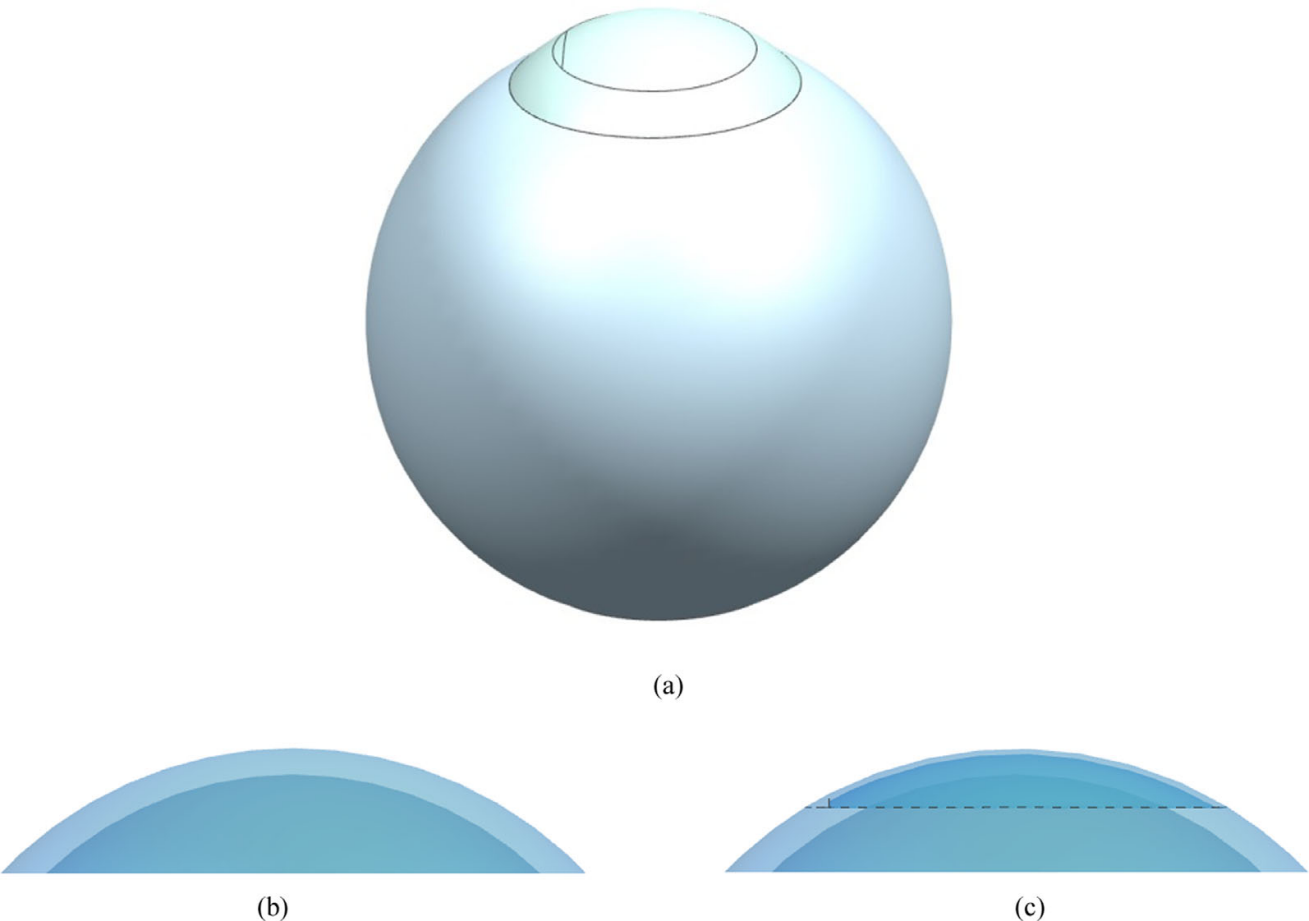
(**a**) The model with myopia diopter of –1 D after conventional laser refractive surgery. (**b**) Corneal perspective view before refractive surgery. (**c**) Corneal perspective view with hinged flap.

According to the ablation profile for pure myopic, different corrected refractions were accompanied by varied thicknesses of substrate ablation. The postoperative corneal flap was obtained by subtracting the ablation depth in the optical zone from the surface of the stromal layer below the corneal flap. A whole-eye model was further simulated by modifying the shape of the anterior corneal surface. The hinged flap was modeled as a crack on the anterior surface of the cornea. The flap started from a specified angle, extending counter-clockwise to the specified angle at a specified radius along the surface. The arc length of the hinge used in each eye was 4 mm. In this study, thickness and diameter of the corneal flap were 100 µm and 8 mm, respectively, and diameter of the optical zone was 6 mm. The range of corrected refractions was –1.0 to –15.0 diopters (D). The corneal surface displacement under IOP was measured. Figure 2 summarizes the myopia –1 D whole-eye model after conventional laser refractive surgery and corneal details before and after refractive surgery.

### Induced Wave-Front Aberrations from Corneal Surface Displacement

During conventional laser refractive surgery, the partial corneal stroma layer is ablated and the corneal thickness decreases, leading to changes in biomechanics effects that affect the corneal shape and alter the refractive state of the cornea. The change of the corneal shape is mainly represented by the displacement of the anterior and posterior surfaces of the cornea, and the change of the refractive state can primarily be observed in the induced wave-front aberrations after conventional laser refractive surgery. In other words, the displacement can be converted into an aberration. The specific analysis proceeds as follows:

a) The displacement (ΔX, ΔY, and ΔZ) of the corneal surface nodes under IOP before and after refractive surgery were obtained from the FEM.
b) The optical path difference of any arbitrary point on the corneal surface before and after IOP loading was calculated from ΔX, ΔY, and ΔZ. Because the cornea is considered to be spherical, the value of Z of any point A (X, Y, Z) on the corneal surface was calculated using the following equation:
  (6) $$Z=\sqrt{R^{2}-X^{2}-Y^{2}}$$

here, R represents the radius of corneal curvature.

Significant corneal deformations were observed under the effect of IOP. At this time, the corresponding point of point A is A' (X+ΔX, Y+ΔY, Z+ΔZ). Then A' corresponds to point B (X+ΔX, Y+ΔY, Z+ΔZ-D) in the Z direction of the corneal surfaces before IOP loading, which can be defined mathematically by the following equations:

(7) $$Z+\Delta Z-D=\sqrt{R^{2}-{\left(X+\Delta X\right)}^{2}-{\left(Y+\Delta Y\right)}^{2}}$$

**Figure 3.**
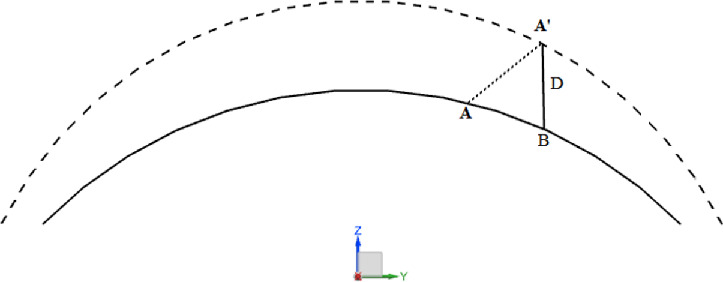
Deformation of the cornea before and after IOP loading. The *black dotted line* indicates the corneal surface under the load of IOP, whereas the *black solid line* depicts the corneal surface without IOP loading.

Schematic diagram of corneal deformation is expressed in Figure 3.

Together with Equations 6 and 7, the displacement D can be calculated

(8) $$D=\Delta Z-\sqrt{(R_{}^{2}\;-\;{\left(X+\Delta X\right)}^{2}\;-\;{\left(Y+\Delta Y\right)}^{2}}\;-\;\sqrt{{R_{}}^{2}\;-\;X^{2}\;-\;Y^{2}}$$

then optical path difference (OPD) can be obtained

(9) OPD=(n-1)D

where *n* is the refractive index of the cornea.

c) Thus we obtained the preoperative Zernike coefficients by wave-front surface fitting from preoperative optical path difference. The same method can be used to obtain the Zernike coefficients after refractive surgery. The induced aberrations were obtained from the differences between the postoperative and preoperative wave-front aberrations, which were estimated as follows:
  (10) $$\begin{array}{ccc} W_{d}\left(x,y\right) & = & W_{post}\left(x,y\right)-W_{pre}\left(x,y\right)\\ & = & \sum_{i=1}^{M}c_{i}Z_{i}\left(x,y\right)-\sum_{i=1}^{M}a_{i}Z_{i}\left(x,y\right) \end{array}$$

here, *c_i_* is the postoperative Zernike coefficient, and *a_i_*is the preoperative coefficient.

First, the relationship between the induced aberrations by the anterior/posterior corneal surface displacement and the corrected refractions was discussed. Induced wave-front aberrations refer to the differences between the postoperative and preoperative induced wave-front aberrations from the corneal surface displacement under the same IOP loading. Second, the relationship between the induced wave-front aberrations from the anterior corneal surface and IOP was studied.

## Results

### Induced Wave-Front Aberrations from Anterior Corneal Surface Displacement

**Figure 4.**
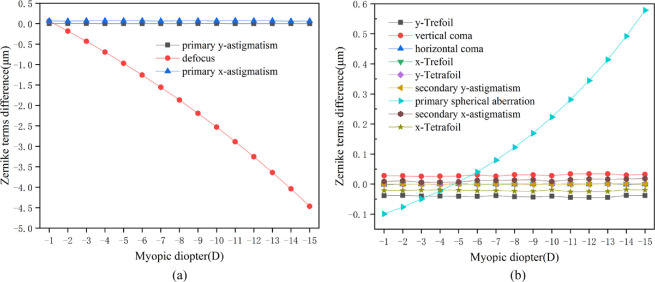
(**a**) Relationship between induced lower-order wave-front aberrations on anterior corneal surface from biomechanical effects and myopic diopter. (**b**) Relationship between induced higher-order wave-front aberrations on anterior corneal surface from biomechanical effects and myopic diopter. The corneal flap thickness was assumed to be 100 µm and the size was 8 mm. IOP was 15 mm Hg. The diameter of the optical zone was 6 mm.

By loading the IOP, the displacement of the anterior and posterior surfaces of the postoperative cornea could be calculated. The induced wave-front aberrations after refractive surgery were computed as the differences between the postoperative and preoperative wave-front aberrations from the displacement of the corneal surface. The corresponding Zernike coefficients were obtained by the surface fitting. Figure 4 shows the curves of the induced aberrations from the biomechanical effects versus the myopic diopter. The diameter of the optical zone was 6 mm.

Overall, the biomechanical effects-induced aberrations after conventional refractive surgery were mainly composed of defocus and primary spherical aberration. According to Figure 4a, when the diameter of the optical zone was 6 mm, the defocus caused by the anterior corneal surface significantly increased with the elevated myopic diopter, and it showed the patterns of a hyperopic shift. The values of primary y-astigmatism and primary x-astigmatism are almost 0. As observed from Figure 4b, the spherical aberration also increased significantly with the increased myopic diopter, which moved from an initial negative value of –0.1 µm to nearly 0.6 µm. Second, the induced aberrations included vertical coma and y-trefoil. However, these two aberration terms remained stable with the increase of myopic diopter, and their high-order aberration terms were relatively minor. The higher-order aberration terms mentioned here mainly included third- and fourth-order aberrations because others were too small to be noticed.

### Induced Wave-Front Aberrations from Posterior Corneal Surface Displacement

**Figure 5.**
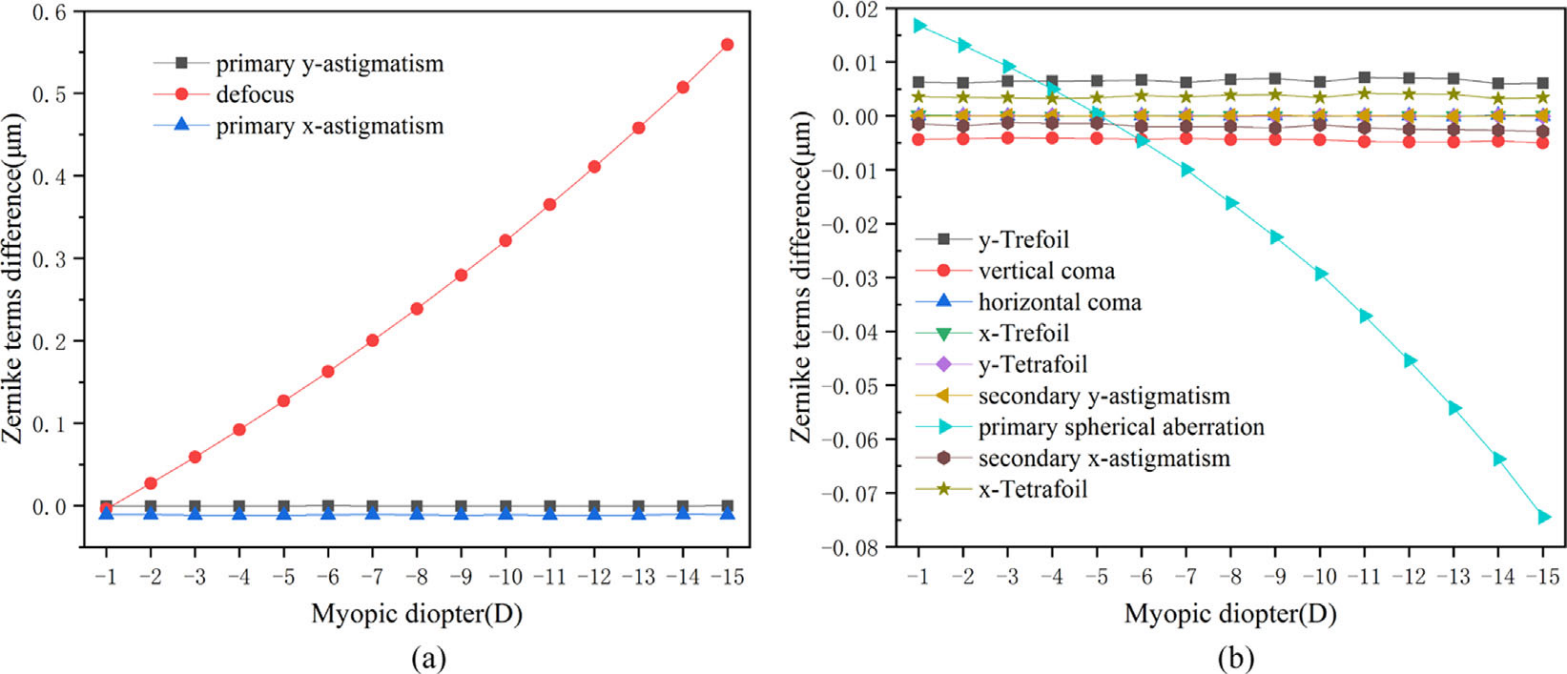
(**a**) Relationship between induced lower-order wave-front aberrations on posterior corneal surface from biomechanical effects and myopic diopter. (**b**) Relationship between induced higher-order wave-front aberrations on posterior corneal surface from biomechanical effects and myopic diopter. The corneal flap thickness was assumed to be 100 µm and the size was 8 mm. IOP was 5 mm Hg. The diameter of the optical zone was 6 mm.

By utilizing the same method, the induced wave-front aberrations on the posterior corneal surface were obtained. Figure 5 shows the curves of the induced aberrations from the biomechanical effects versus the myopic diopter. The diameter of the optical zone was 6 mm.

Overall, the biomechanical effects-induced aberrations after conventional laser refractive surgery were mainly composed of defocus and primary spherical aberrations, which was consistent with the results of the anterior corneal surface. However, the introduced high-order aberrations were nearly an order of magnitude lower than those of the anterior surface. According to Figure 5a, when the diameter of the optical zone was 6 mm, the defocus caused by the posterior corneal surface was most impacted by the myopic diopter, and it showed the patterns of a myopic shift. According to Figure 5b, the primary spherical aberration was most affected by the myopic diopter and shifted from a positive value to a negative value as the myopic diopter increased. Vertical coma and y-trefoil remained unchanged with the enhanced myopic diopter. Most importantly, the aberrations introduced by the posterior surface of the cornea was less affected than that introduced by the anterior surface. However, a partially compensatory mechanism between the induced wave-front aberrations from the posterior corneal surface and those from the anterior corneal surface was also demonstrated.

### Effect of IOP on Induced Wave-Front Aberrations from Anterior Corneal Surface Displacement

**Figure 6.**
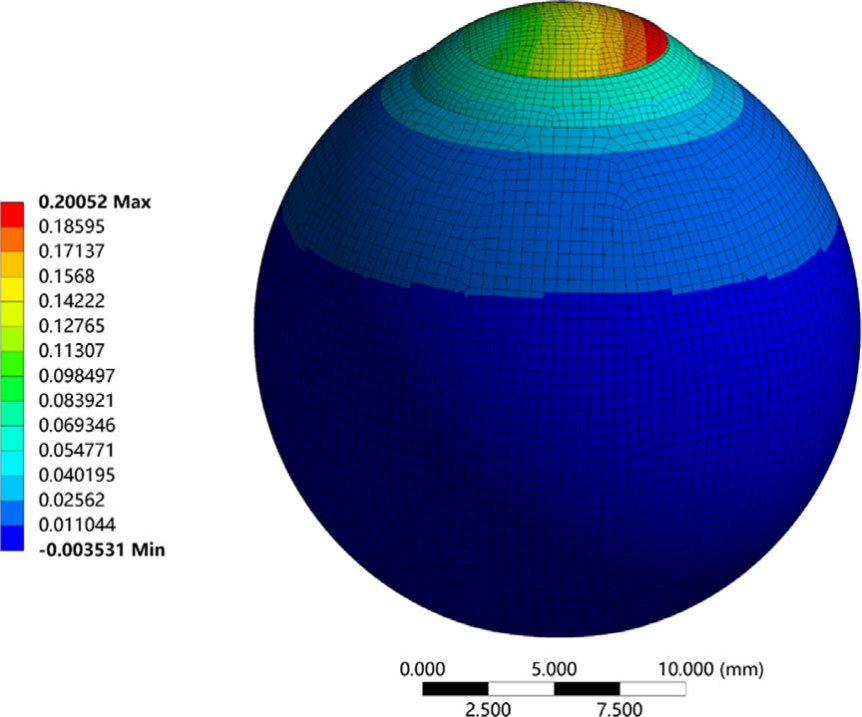
Displacement distribution of the human eye in the Z direction as the IOP was 15 mm Hg.

The corneal surfaces displacement could be obtained from the FEM under IOP. In fact, the IOP had a significant influence on corneal surface displacement. In addition, clinically, IOP varies greatly among individuals. Therefore it deserves great attention to further study the relationship between IOP and wave-front aberrations of anterior corneal surface. First, the nephogram of corneal deformation is shown in Figure 6 as IOP being 15 mm Hg.

**Figure 7.**
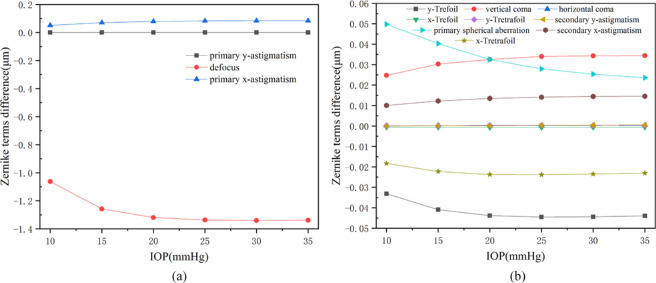
(**a**) Relationship between induced lower-order wave-front aberrations on anterior corneal surface from biomechanical effects and IOP. (**b**) Relationship between induced higher-order wave-front aberrations on anterior corneal surface from biomechanical effects and IOP. The corrected refraction was set to be –6 D. The corneal flap thickness was assumed to be 100 µm and the size was 8 mm. IOP was 15 mm Hg. The diameter of the optical zone was 6 mm.

As indicated by Figure 6, the incision of the corneal flap created a larger displacement on the right side, which was mainly caused by the separation of the corneal flap layer and the stromal layer. However, this phenomenon was not clearly apparent after the actual surgical procedure. In this part, the association between the induced aberrations and IOP in the range from 10 to 35 mm Hg was investigated. For the corrected refraction of –6 D, the results are shown in Figure 7.

It was concluded from Figure 7 that when the corrected refraction was –6 D, the defocus decreased slightly and tended to be stable with the increase of IOP. The vertical coma increased as the IOP increased, whereas the primary spherical aberration decreased with the increase of IOP. In fact, in clinical cases, the statistical results had shown that the IOP after refractive surgery was less than the preoperative value. Here, we did not consider the change of IOP before and after surgery.

## Discussion

### Comparison with Previous Studies

Refractive surgery changes the shape of the anterior corneal surface, which leads to changes in corneal refractive power and corrections in refraction errors.^21^^,^^22^ However, clinical studies have shown that the higher-order aberrations of the eyes significantly increase after refractive surgery, partly due to biomechanical effects of the cornea. Our study has shown that the higher-order aberrations caused by the displacement of the anterior and posterior corneal surfaces after refractive surgery mainly include spherical aberration, vertical coma, and y-trefoil, which is consistent with the clinical data published by Wu and Wang.^23^ In addition, Benito et al.^24^ found that positive spherical aberration and coma were associated with a similar increase in corneal aberrations after myopic LASIK. Therefore the higher-order aberrations after LASIK partly resulted from the biomechanical effects of cornea.

Deenadayalu et al.^7^ showed that patient eyes (simulated) with an elastic cornea (modulus of elasticity = 2 MPa) could undergo a hyperopic shift as large as 2.0 D with just the introduction of the flap. However, in our study, the cornea ablation resulted in a 0.75 D hyperopic shift on refractive correction to –5 D. The different results may be due to differences in materials and models used in these studies.

Fang et al.^20^ analyzed the impact of refractive surgery on wave-front aberrations and found that significant higher-order aberrations were induced by the ablation profile based on a mathematical model of the anterior corneal surface. Therefore we concluded that these postoperative higher-order aberrations may be caused by the combination of laser ablation to the stromal bed and the anterior and posterior corneal surface displacement associated with the biomechanical weakness of the cornea.^15^

### Induced Wave-Front Aberrations from Anterior Corneal Surface Displacement with Treatment Decentration

**Figure 8.**
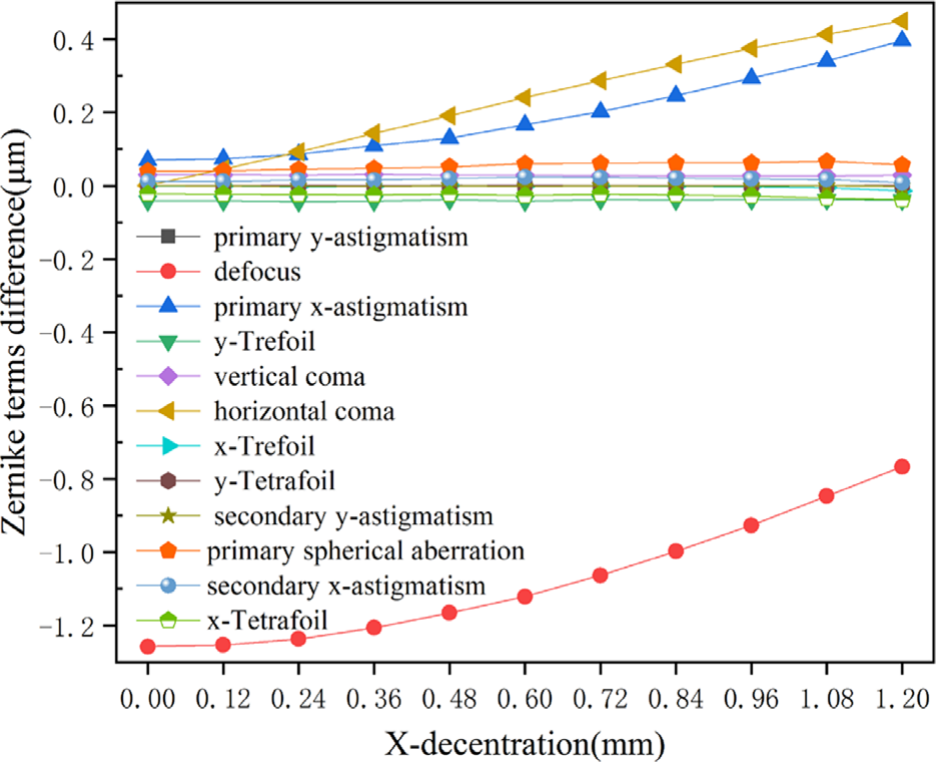
The relationship between induced wave-front aberrations from biomechanical effects on anterior corneal surface and treatment decentration. The flap thickness was set at 100 µm. The myopic diopter was set at –6 D and IOP was set at 15 mm Hg. The diameter of the optical zone was 6 mm.

In clinical surgery, the center of the pupil changes with different light conditions and sitting positions during check and surgery, resulting in eccentric laser ablation. Therefore analysis of the aberrations caused by anterior corneal surface displacement from treatment decentration has clinical significance. Figure 8 showed the aberrations caused by the biomechanical effects with different levels of treatment decentration on the X-axis after the correction of –6 D myopia.

Increased treatment decentration on the X-axis was associated with a decreased hyperopic shift and significantly increased primary x-astigmatism. Moreover, treatment decentration resulted in increasing in higher-order aberrations, mainly coma. Treatment decentration on the X-axis led to horizontal coma.

### Induced Wave-Front Aberrations from Anterior Corneal Surface Displacement at Large Pupil Size

**Figure 9.**
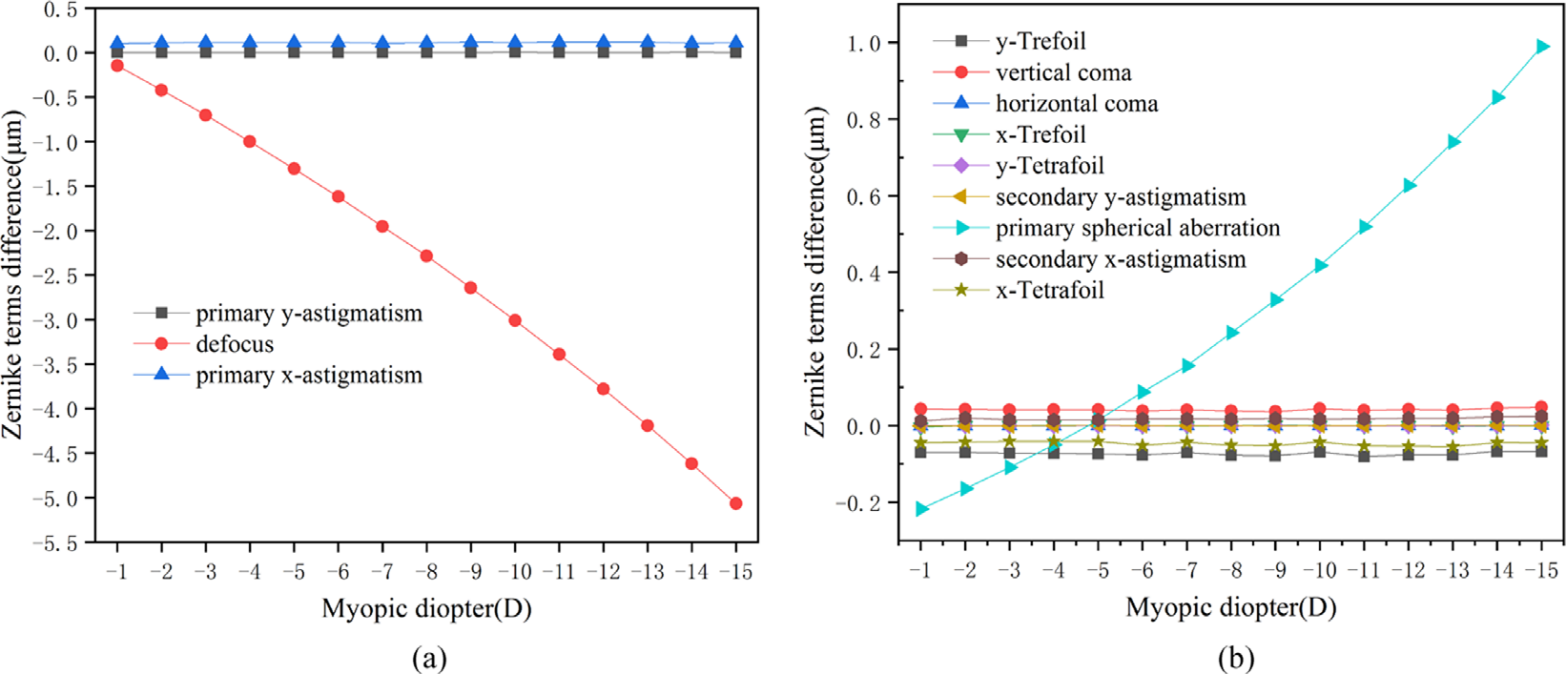
(**a**) Relationship between induced wave-front aberrations from biomechanical effects on anterior corneal surface and myopic diopter. (**b**) Relationship between induced higher-order wave-front aberrations from biomechanical effects on anterior corneal surface and myopic diopter.

The size of the pupil greatly affects the root-mean-square (RMS) of wave-front aberrations. Considering the impacts of the biomechanical effects, a larger pupil diameter significantly contributes to the higher magnitude of aberrations. Similarly, the displacement of the anterior corneal surface would induce wave-front aberrations. The induced wave-front aberrations were computed as the differences between the postoperative and preoperative wave-front aberrations from the displacement of the anterior corneal surface. The corresponding Zernike coefficients were obtained by surface fitting. Figure 9 shows the curves of the induced aberrations from the biomechanical effects versus the myopic diopter. The diameter of the optical zone was 6 mm, but the diameter of the pupil was set as 7 mm.

Compared with Figure 4, the aberration structure of 7-mm pupils was similar to that of 6-mm pupils after refractive surgery. However, the aberrations in 7-mm pupils were larger than those in 6-mm pupils. This was mainly because the RMS values of wave-front aberrations were closely associated with pupil size, with larger pupils having higher RMS values. This finding was in line with previous studies, which had also shown that larger pupils were associated with greater contributions to spherical aberration following surgery.^25^

### Induced Wave-Front Aberrations from Anterior Corneal Surface Displacement with the Effect of Material Parameters of the Ocular Tissue

Material parameters are key to biomechanical response of biological tissue. Previous studies have also demonstrated that the material parameters of cornea and sclera vary greatly among individuals.^26^ Therefore effect of material parameters of the ocular tissue on wave-front aberrations from anterior corneal surface was studied. At present, some studies have suggested that the Young modulus of the sclera was approximately 3 to 5 times higher than that of the cornea.^27^ In addition, it is worthwhile to consider the range of the elastic modulus of the cornea in normal human eyes, so eight cases were designed to support the effect of material parameters on the introduction of aberrations as shown in Table 1.

**Table 1.** Summary of Mechanical Properties of Ocular Tissues

	Cornea			Sclera		
Case	µ_1_	α_1_	Elasticity E (MPa) as IOP = 15 mm Hg	µ_1_	α_1_	Elasticity E (MPa) as IOP = 15 mm Hg
1	0.0084	13.312	0.108	0.0254	23.716	0.325
2	0.0109	32.192	0.325	0.0289	63.49	0.974
3	0.0120	88.684	0.974	0.0302	182.73	2.923
4	0.0124	258.09	2.923	0.0307	540.4	8.770
5	0.0114	46.321	0.487	0.0302	182.73	2.923
6	0.0120	88.684	0.974			
7	0.0123	173.39	1.949			
8	0.0125	342.8	3.898			

In this study, the material model was nonlinear, the Young modulus of the cornea and sclera in the table was actually obtained as IOP being 15 mm Hg. From case 1 to case 4, the elastic range of the cornea was from 0.1 to 3 MPa, and the elastic range of the sclera was from 0.3 to 8 MPa. From case 5 to case 8, the material parameters of the sclera maintained a constant, whereas that of the cornea was in the range from 0.49 to 3.9 MPa. These cases corresponded to the ratio of corneal and scleral material parameters in clinical practice. In Table 2, the induced wave-front aberrations from the anterior corneal surface displacement with the corrected refraction of –6 D are shown for the eight cases.

**Table 2.** Summary of Induced Zernike Aberrations on the Anterior Cornea

	Wave-Front Aberration									
	Low-Order Aberration (µm)		High-Order Aberration (µm)							
Case	C_22_	C_20_	C_33_	C_31_	C_44_	C_42_	C_40_	C_55_	C_53_	C_51_
1	0.167	−6.016	0.010	0.082	0.052	0.042	0.069	0.011	0.004	0.011
2	0.104	−2.870	0.005	0.053	0.038	0.026	0.104	0.015	0.006	0.001
3	0.051	−1.146	0.001	0.025	0.019	0.011	0.064	0.008	0.002	0.002
4	0.020	−0.414	0.000	0.010	0.007	0.004	0.028	0.003	0.001	0.001
5	0.082	−2.028	0.056	0.043	0.031	0.019	0.075	0.013	0.004	0.001
6	0.051	−1.146	0.034	0.025	0.019	0.011	0.064	0.008	0.002	0.002
7	0.028	−0.647	0.019	0.013	0.011	0.006	0.051	0.005	0.001	0.001
8	0.014	−0.372	0.010	0.006	0.005	0.003	0.036	0.002	0.000	0.001

According to Table 2, the material parameters of ocular tissues had a significant influence on hyperopic shift. However, the effects on higher-order aberrations were relatively small, and the induced aberrations were less than 0.11 µm. If the elastic modulus of the cornea and sclera increased simultaneously, the low-order aberrations after refractive surgery decreased, especially the hyperopic shift. When scleral elasticity remained constant, corneal elasticity increased, the hyperopic shift and spherical aberration decreased. This suggested that the changes in the corneal elasticity would affect the postoperative spherical aberration, but to a lesser extent than expected.

### Other Thoughts

This study assessed the impact of the biomechanical effects of human eye on the wave-front aberrations after refractive surgery based on FEM. A quantitative approach was used to analyze the relationships between the induced aberrations from the biomechanical effect and myopic diopter or IOP. The induced aberrations were calculated from FEM models by focusing on the corneal deformation from the biomechanical effects. However, the Gullstrand classic eye model and the Munnerlynn-based profile was used, which did not consider the individual clinical data, and only wave-front aberrations induced from the biomechanical effect were considered in our research. This was a major limitation of the study. Furthermore, the residual wave-front aberrations from clinical measurements may be derived from the ablation profile, treatment decentration, wound healing, and dynamic characteristics of eye aberrations and also other factors. In addition, another limitation was that corneal asphericity and the epithelial remodeling was not considered, which may be a possible source of postoperative aberrations and further study is needed to clarify this issue. Therefore our results were not completely consistent with clinical data.

In this study, the corneal displacement included not only the displacement of the anterior and posterior corneal surfaces through the Z-axis, but also the displacement through both the X- and Y-axis, making the calculation of wave-front aberrations more accurate.

Our analysis found that the corneal flap induced coma, and the value of coma was associated with the position and shape of the connection, but not with the corrected refraction. However, without the impact of the corneal flap, only few comas were induced. In addition, there are some other asymmetric aberrations with smaller values, which can be attributed to nonrotational symmetry of surface fitting and meshing.

Our analysis also found that the material parameters of the corneal and scleral tissues played an important role in their biomechanical and optical behavior.^28^ The material parameters of the cornea significantly affect the displacement of the anterior and posterior corneal surfaces. Moreover, once the scleral elasticity maintained a constant, with the increase of corneal elasticity, the maximum stress and maximum displacement moved toward the edge of the cornea. This finding was consistent with the studies published by Roy and Dupps.^6^ Therefore further studies should focus on the effect of this result on the induced aberrations after refractive surgery. In addition, with the fixed material parameters of the cornea, the scleral material parameters still significantly affected the shape of the cornea. The material parameters of both the cornea and the sclera in this study were from previous publications. However, previous studies had shown significant individual differences in the corneal material parameters. The individual corneal material parameters was obtained by fitting a material model to experimental data of corneal tissue using the inverse finite element approach, but it had not been applied in clinical practice.^29^ In fact, the proper in vivo measurement of the material parameters of cornea and sclera was required for the construction of a more precise and individual human eye FEM.^30^

In addition, the factors affecting the biomechanical response after refractive surgery are too many. First, one factor is the refractive surgery procedures. Seven et al.^31^ found that higher deformations and stresses were observed within the residual stromal bed in flap-based cases than SMILE cases. In the Sinha Roy et al.^32^ work, SMILE may present less biomechanical risk in the corneal residual bed than comparable corrections involving LASIK flaps. Second, another factor is the microstructure of the corneal tissue, such as the local micromechanical properties of different layers in the cornea,^33^ and the distribution of physiological collagen fibers exhibiting nonlinear anisotropy.^34^ Finally, the factor is the large differences among individuals in biomechanical property. The accuracy of simulation-based LASIK outcomes could be improved by the establishment of patient-specific simulation.^35^

Based on finite element analysis, the development of an individual eye model can help improve understanding of the biomechanics of the eye.^36^^,^^37^ Future research finding would be closer to clinical measurement data by using the ablation profiles for different surgical procedures and the individual eye models. Our goal is to simulate the clinical situation as much as possible. In follow-up work, the effect of refractive surgery on the biomechanical properties may be better evaluated by constructing individual FEM of the human eye combined with treatment decentration, transition zone, corneal flap, optical zone size, IOP, and other parameters.^38^ In addition, we would also focus on the data of stress and strain in the results of the finite element analysis to better understand the biomechanical characteristics of the human eye. Finite element method can become a valuable tool to plan and design refractive surgery^39^ and other ophthalmo-surgical procedures to optimize the refractive outcomes and the visual function.^40^

## Conclusions

Using the human eye FEM to simulate conventional refractive surgery, we found that the corneal biomechanical effects resulted in significant changes in the anterior and posterior corneal surfaces and wave-front aberrations were induced. Our results showed that the anterior corneal surface displacement led to the hyperopic shift after refractive surgery. Especially with the increase of the corrected refraction, the hyperopic shift also increased. Higher corneal tissue ablation resulted in bigger biomechanical effects. The induced higher-order aberrations mainly included spherical aberration, vertical coma, and y-trefoil. The spherical aberration increased with the increases of the corrected refraction, whereas the change of the corrected refraction did not affect the vertical coma and y-trefoil. The induced aberrations from the corneal posterior surface displacement were much smaller than those of the anterior surfaces. IOP had a slight effect on the induced aberration after refractive surgery. With the increase of IOP, the hyperopic shift increased and tended to be stable, whereas the coma increased, and the spherical aberration decreased. Treatment decentration mainly affected coma and spherical aberration. The value of coma increased with the increase of treatment decentration. The induced aberrations were also affected by the material parameters of the ocular tissue. For example, when the scleral elasticity was constant and the corneal elasticity was increased, the induced aberrations decreased. The analysis based on FEM revealed that the biomechanical effects after refractive surgery were one of the main contributors to increases in residual wave-front aberrations. Therefore the biomechanical effects of the human eye should be considered in the design and analysis of refractive surgery.
